# The Annual Address, Delivered before the New York State Medical Society, and the Members of the Legislature, at the Capitol, Feb. 1853

**Published:** 1853-08

**Authors:** 


*The Annual Address, delivered before the New York State Medical Society, and the Members of the Legislature, at the Capitol, Feb.* 1853. By A. Clark, M. D., President of the Society.

It is a pity that productions of this kind cannot find some more effective way of reaching the people than the pamphlet form. The medical profession knows the whole story of its wrongs, its sacrifices, and its holy devotion to the cause of scientific truth. Dr. Clark, in this address, has presented them in a most forcible style. It is an elegantly written discourse; and if Dr. Clark, or some one else, would publish an equally good one in the columns of some one of our widely circulated literary magazines, he would merit, (even more than now,) the thanks of the profession. We give the the following extract from the address:

"Thus I have attempted to show what medicine has done, and by inference what it can do; the means by which it has made this progress, and the motives that have excited it to exertion. An important inference naturally flows from what has been said. That the condition of the medical profession is the personal concern of every member of society, consequently, a vast public interest.

"A virtuous and conscientious course of conduct brings with it its own reward, doubtless. Learning, by the lively gratification its acquisition affords, will compensate many a man for great exertions in its pursuit. Charity doubtless blesses the giver, whatever may be its influence on the recipient of the intended benefit; yet who but an utopian would trust a great enterprise to these influences alone? In all associations of men, from the school-house to the senate chamber; in all the relations of society, uprightness expects and receives approbation, as no inconceivable part of the reward; learning commands respect, perhaps even reverence; charity, esteem and love. Is society then just to itself, when it leaves the great work of the profession to go on without its commendation, without its notice? I say *to go on*, for go on it will. No neglect can stay its progress. There is a thirst for learning in him who has assumed the high duties of a conscientious physician, which deep drafts alone can slake. There is an '*esprit du corps*' which has risen, and will rise superior to neglect, even to ridicule and detraction. Yet I ask again, is society just to itself, when it leaves to the unencouraged labors of a body of men in a great degree isolated, interests in which every man has so large a stake. Let me step a little out of my course, for an illustrative contrast:

"On the 2d of April, nearly five years ago, the city of New York was thrown into mourning by the news that two firemen were suddenly killed in the performance of their duty. The next day the common council was convened, to give expression to this sense of sorrow. The memory of these men was honored with a public funeral. Sermons were preached in commemoration of their virtues and courage, and the city charged itself with the education of their fatherless children. This was as it should be. Whenever personal dangers are to be met for the benefit of others, necessary exposure should be encouraged by extraordinary rewards.

"Some who hear me will remember the pageant of the succeeding 12th of July. The sun of that day rose on the national flag at half-mast over all the public buildings of that city. He who passed through the park on the morning of that day, might have seen a platform there shrouded in black, which, though unoccupied, was already surrounded by a solemn, silent multitude. The citizen soldiery was at the same time mustering from every part of the town, for honors had been decreed to the brave, who had fallen in the land of the stranger. Then came the gorgeous procession with crape-clad banners, marching slow to solemn music, and the church-bells tolled funeral knells as it passed by. A hundred thousand people read with saddened hearts, on the black drapery of the hearses, the names of the fallen. For three hours that pompous procession marched by, proclaiming that the soldier shall be honored in his death. Then that sombre platform was filled. There sat the city council; there were men old and honored in the public service; and that hushed and solemn multitude, now increasing by thousands, stood gazing on the coffins of the dead. The orator pronounced the eulogy of the fallen, and expressed the sympathy of the popular heart; the minister of the gospel added Christian benediction; and thus ended, in the language of a newspaper, 'a scene of mourning and solemn pageantry, such as was never before witnessed in this great metropolis.' Thus, then, did glory pay its debt to valor.

"That same year another little body of soldiers might have been seen, here at home, making defensive war against an invading enemy. One after another of that little band sunk mortally wounded on *their* field of battle; and when at length they were gathered to their graves, glory awoke no pæans for them. The public heart, cold as the earth which received their mortal remains, felt no pang. Alas! out of the desolated family circle — out of the profession that cherishes their memories — who was there to mourn for Snowden? Who was there to mourn for Graham? Who was there to mourn for Beals, Hutchinson, Porter, Van Buren, Hedges, Blakeman, Calhoun, Worth and Leonard? Yet these were among the men who stood between the living and the dead; these were the costly sacrifice, and yet not all the sacrifice, by which the *fever pestilence* was stayed. And have they died in vain?

"But for the conscience that urges us to the performance of duty at every risk; but for the spirit of martyrdom,— I do not speak too strongly, though I say it proudly,— but for the spirit of martyrdom, which the profession inculcates, and which the performance of its duties ripens; and above all, but for that High Court of Appeals, which must so often reverse man's judgment, presided over by Him who hath said: 'Greater love hath no man than this, that a man lay down his life for his friends;' but for these things, they had died, perhaps, in vain. Yet they fell at their posts, surrounded by known and appreciated dangers; the soldier could do no more. May I not ask, then, disclaiming all desire to underrate the soldier, or to abate one tittle of his high repute among men, may I not ask the soldier himself, in what the conscientious physician is less deserving than he? Is the soldier less selfish? Disinterestedness will hardly be claimed as the ruling motive with either. Does he show a more exalted patriotism? He destroys the enemies of the republic; the physician saves the lives of its friends. The soldier's glory is his valor; and yet show me, on all the emblazoned chronicles of his most brilliant darings, acts of more intrepid courage than grace the modest records of our profession. The pestilence strikes terror to the heart of every man; the physician never turns from it. From the dreadful days when death grew frantic with its own work of slaughter, and Hippocrates stood up to wrestle with it night and day in terror-stricken Athens, to the hour when the affrighted people of our own time fled before the most appalling of all plagues that ever scourged the earth, the physician has never turned his back on danger. Where the sufferer calls, though the way to him and the air about him are laden with the breath of the pestilence, yet he never calls in vain. I have known the soldier of twenty battles turn pale, and flee before the least of the physician's perils. I well remember when, twenty years since, the first blow of this last and most relentless of death's agents fell on the city in which I dwell, how the heart of the brave man became as the heart of the child, how the very name of pestilence blanched every cheek, how thousands on thousands fled, till in ten days the town was emptied of half its inhabitants. 'Save himself who can,' was the principle of the time. Even the ministers of the gospel, one denomination excepted, sought safety in flight. Yet the ranks of the profession were well nigh unbroken. Out of more than five hundred physicians, I could hear of but three fugitives; and out of eighty medical students, then in the city, I could not learn that there was one. I can tell you of a hundred posts of danger where numbers fall annually, and yet their places are immediately supplied by others equally courageous. Of the Irish physicians engaged in hospital practice, one-half sicken with fever, and one-sixth die of it; yet the poor in these institutions always have the best medical services in the country rendered gratuitously. Of thirty assistant physicians doing duty at the Bellevue Hospital in New York, during the late prevalence of ship-fever in that city, twenty-one took the disease, and five died of it; and even of the nine who escaped it there, three had already suffered from it in other medical institutions. Yet their ranks were always full; and I speak from personal knowledge, when I say that I know not where to look for a body of young men whose duty is performed with more conscientiousness, and courage, and intelligence."

